# Leaf transcriptome analysis of a subtropical evergreen broadleaf plant, wild oil-tea camellia (*Camellia oleifera*), revealing candidate genes for cold acclimation

**DOI:** 10.1186/s12864-017-3570-4

**Published:** 2017-02-28

**Authors:** Jiaming Chen, Xiaoqiang Yang, Xiaomao Huang, Shihua Duan, Chuan Long, Jiakuan Chen, Jun Rong

**Affiliations:** 1 Center for Watershed Ecology, Institute of Life Science and School of Life Sciences, Nanchang University, Nanchang, 330031 Jiangxi Province China; 2 Key Laboratory of Poyang Lake Environment and Resource Utilization, Ministry of Education, Nanchang University, Nanchang, 330031 Jiangxi Province China; 3 School of Life Sciences, Jinggangshan University, Ji’an, 343009 Jiangxi Province China; 4 Jinggangshan National Nature Reserve Administration Bureau, Jinggangshan, 343600 Jiangxi Province China

**Keywords:** *Camellia oleifera*, Cold acclimation, Differential gene expression, Genetic structure, Molecular marker, Transcriptome, Wild oil-tea camellia

## Abstract

**Background:**

Cold tolerance is a key determinant of the geographical distribution range of a plant species and crop production. Cold acclimation can enhance freezing-tolerance of plant species through a period of exposure to low nonfreezing temperatures. As a subtropical evergreen broadleaf plant, oil-tea camellia demonstrates a relatively strong tolerance to freezing temperatures. Moreover, wild oil-tea camellia is an essential genetic resource for the breeding of cultivated oil-tea camellia, one of the four major woody oil crops in the world. The aims of our study are to identify variations in transcriptomes of wild oil-tea camellia from different latitudes and elevations, and discover candidate genes for cold acclimation.

**Results:**

Leaf transcriptomes were obtained of wild oil-tea camellia from different elevations in Lu and Jinggang Mountains, China. Huge amounts of simple sequence repeats (SSRs), single-nucleotide polymorphisms (SNPs) and insertion/deletions (InDels) were identified. Based on SNPs, phylogenetic analysis was performed to detect genetic structure. Wild oil-tea camellia samples were genetically differentiated mainly between latitudes (between Lu and Jinggang Mountains) and then among elevations (within Lu or Jinggang Mountain). Gene expression patterns of wild oil-tea camellia samples were compared among different air temperatures, and differentially expressed genes (DEGs) were discovered. When air temperatures were below 10 °C, gene expression patterns changed dramatically and majority of the DEGs were up-regulated at low temperatures. More DEGs concerned with cold acclimation were detected at 2 °C than at 5 °C, and a putative C-repeat binding factor (CBF) gene was significantly up-regulated only at 2 °C, suggesting a stronger cold stress at 2 °C. We developed a new method for identifying significant functional groups of DEGs. Among the DEGs, transmembrane transporter genes were found to be predominant and many of them encoded transmembrane sugar transporters.

**Conclusions:**

Our study provides one of the largest transcriptome dataset in the genus *Camellia*. Wild oil-tea camellia populations were genetically differentiated between latitudes. It may undergo cold acclimation when air temperatures are below 10 °C. Candidate genes for cold acclimation may be predominantly involved in transmembrane transporter activities.

**Electronic supplementary material:**

The online version of this article (doi:10.1186/s12864-017-3570-4) contains supplementary material, which is available to authorized users.

## Background

Cold tolerance is a key determinant of the geographical distribution range of a plant species and crop production [1–3]. Many plant species demonstrate an increase in freezing tolerance upon a period of exposure to low nonfreezing temperatures, a phenomenon known as cold acclimation [4]. In nature, the process of cold acclimation helps plants to prepare for the coming of winter so as to survive under seasonal freezing temperatures. On the other hand, freezing damage on crops can lead to serious loss of crop production [3]. Therefore, intensive researches have been conducted in many plant species for understanding the molecular mechanisms of cold acclimation [1, 2]. The majority of the previous studies focused on herbaceous plant species (e.g. Arabidopsis). A review by Wisniewski et al. [3] indicated that the molecular mechanisms of cold acclimation in woody plants were more complex than in herbaceous plants, and it was still not clear about what made a woody plant more cold hardy than an annual, herbaceous plant.

As an evergreen broadleaf shrub or small tree, oil-tea camellia (*Camellia oleifera*) is one of the representative plant species in subtropical evergreen broadleaf forests [5] with relatively strong tolerance to cold climates. Oil-tea camellia is widely distributed in the subtropical mountain areas of the Yangtze River basin and South China, with elevation ranging from about 200 to 2000 m [5, 6]. The northern range of oil-tea camellia is located in the mountain areas of the north subtropical region in China, where the mean annual air temperature is 14–16 °C, the mean January air temperature is 0–4 °C, and the minimum air temperature is as low as −17 °C [6]. It has been reported that oil-tea camellia could survive under −26 °C in the USA, and was used to cross with *C. sasanqua* and *C. hiemalis* (susceptible to winter injury) for producing ornamental camellia varieties with cold tolerance [7, 8]. As an evergreen broadleaf plant species, oil-tea camellia has green leaves even in cold winter. Unlike most of the flowering plant species in the world, it flowers in autumn and winter. The fatty acid contents in oil-tea camellia seeds showed significant correlations with latitudes [9], which may be due to natural selection on seed germination temperature [10]. Therefore, oil-tea camellia can be used as a model to study the molecular basis of cold tolerance in evergreen broadleaf plant species. However, the candidate genes related to cold acclimation and the gene expression patterns are still unknown in oil-tea camellia.

Cultivated oil-tea camellia is regarded as one of the world’s four major woody oil crops together with oil palm, coconut and oil olive, and is the top one woody oil crops in China [6]. The utilization of oil-tea camellia seed oil (camellia oil) as cooking oil has a history of more than 2300 years in China [6]. Camellia oil is rich in unsaturated fatty acids (more than 80% of total oil content), containing mainly monounsaturated fatty acid (i.e. oleic acid, contributing to more than 68% of total oil content) and some polyunsaturated fatty acid (i.e. linoleic acid and linolenic acid) [9, 11]. Its fatty acid composition is similar to olive oil, and it is therefore known as “oriental olive oil” [11, 12]. Camellia oil also contains other functional components such as camellia saponin, tea polyphenol and squalene [12]. It has been shown that the intake of camellia oil is good for health, for instance, helping to reduce blood lipid and prevent cardiovascular diseases [12]. Currently, China has about 3 million hectare cultivated oil-tea camellia, producing about 0.26 million ton camellia oil per year [13]. To meet the rapidly increasing demands for healthy vegetable oil, the Chinese government plans to increase the cultivation of oil-tea camellia to more than 4 million hectare by 2020, with a yearly camellia oil production up to 2.5 million ton [13]. The key issues for the development of oil-tea camellia cultivation are how to accelerate the breeding processes of varieties suitable for various regions, increase the yield and quality of camellia oil, and improve the resistance to diseases and pests.

Crop wild relatives are valuable genetic resources for crop breeding, for instance, helping to improve disease and pest resistances, and increase yield and quality of crops [14]. Wild oil-tea camellia (*C. oleifera*) is an essential genetic resource for cultivated oil-tea camellia breeding. However, the patterns of genetic differentiation along latitude and elevation gradients in wild oil-tea camellia are still unknown, which is the basis for the utilization of wild oil-tea camellia resources. Currently, the evaluation of oil-tea camellia genetic resource for selective breeding is based on phenotypic traits. Due to the complex interactions between genotype and environment, phenotypic traits may not reflex the actual level of genetic variation in a population. On the other hand, as a perennial woody plant, oil-tea camellia has a juvenile phase of about 5 years [6]. Many valuable economic traits need to be evaluated in the adult phase, such as fruit and seed characteristics, leading to a traditionally long breeding process of cultivated oil-tea camellia. Therefore, a huge amount of molecular markers shall be developed and applied for the purposes of genetic resource evaluation and marker-assisted breeding, so as to dramatically accelerate the breeding processes. Xia et al. [15] published the first transcriptome sequencing dataset of oil-tea camellia, providing the major genetic information of this species in public databases. However, their study used samples from a single oil-tea camellia individual in the botanic garden, and so the genetic variations were underestimated and could not represent the genetic differentiation along latitude and elevation gradients in nature [15].

Our study sequenced the leaf transcriptomes of wild oil-tea camellia from different latitudes and elevations, and analyzed the variations in gene sequences and gene expressions. The objectives of our study were to: 1) obtain the leaf transcriptomes of wild oil-tea camellia for functional genomics studies; 2) detect simple sequence repeats (SSRs), single-nucleotide polymorphisms (SNPs) and insertion/deletions (InDels) suitable for analyzing genetic differentiations along latitude and elevation gradients in wild oil-tea camellia; and 3) compare the gene expression patterns among different temperatures in leaves of wild oil-tea camellia and discover the candidate genes related to cold acclimation.

## Methods

### Study sites and sampling

Wild oil-tea camellia samples were collected from different elevations in Lu Mountain (29 Nov 2013) and Jinggang Mountain (6 Dec 2013) in Jiangxi Province, China (Table 1). The Lu Mountain is located in the northern range of oil-tea camellia distribution. At the sampling sites of Lu Mountain, the mean annual precipitation of different elevations ranges from 1728 to 1826 mm, the mean annual air temperature is 13.2–14.9 °C, and the lowest air temperature in the coldest month is −1.9–0.5 °C. The Jinggang Mountain is located in the center of oil-tea camellia distribution. The sampling sites at different elevations in Jinggang Mountain have mean annual precipitation of 1553–1719 mm, mean annual air temperature of 14.6–16.9 °C, and the lowest temperature in the coldest month of 0.0–1.7 °C. The mean annual precipitation is higher in the sampling sites of Lu Mountain than in Jinggang Mountain, and the lowest temperature in the coldest month is lower in Lu Mountain than in Jinggang Mountain. Mean annual precipitation and the lowest temperature in the coldest month are the limiting factors for the geographical distribution of oil-tea camellia, and differences in such climate factors may lead to genetic differentiation of wild oil-tea camellia between the two mountains.

**Table 1:** Wild oil-tea camellia samples of different latitudes and elevations for the transcriptome sequencing

Location	Sample^a^	Latitude (N)	Longitude (E)	Elevation (m)	Temperature (°C)^b^	Temperature group
Lu Mountain	LS04	29.588683°	115.984928°	778	2.0	T2
	LS03	29.598854°	115.987218°	469	5.5	T5
	LS02	29.601352°	115.987723°	412	11.0	T10
	LS01	29.609445°	115.981936°	171	9.8	
Jinggang Mountain	JG04	26.555483°	114.153718°	860	14.3	T14
	JG03	26.555263°	114.153966°	856	14.0	
	JG02	26.520661°	114.195885°	400	18.7	T18
	JG01	26.520661°	114.195885°	397	18.2	

^a^RNAs extracted from two leaves of the same plant were equimolarly pooled and used as a single sample for the transcriptome sequencing

^b^Air temperatures next to the sampling plants at the same time of sampling

In each mountain, leaf samples were collected from flowering wild oil-tea camellia at different elevations within 3 h in the afternoon. Three to five fresh leaves without obvious damage were randomly picked from each plant, covered by aluminum foil and immediately placed in a vacuum bottle with liquid nitrogen. Latitude, longitude and elevation of each sampling plant were recorded. At the same time, air temperature next to each sampling plant was measured. All samples were stored at −80 °C in the lab.

### RNA extraction and transcriptome sequencing

Each leaf was mixed with liquid nitrogen and ground into fine powder. About 100 mg tissue powder of each leaf was used for RNA extraction. Total RNAs were extracted using the EASYspin Plus Plant RNA Kit (Aidlab, Beijing, China). To account for the gene expression variations among leaves of the same plant, the RNAs from two leaves of the same plant were equimolarly pooled and used as a single sample for the transcriptome sequencing. In total, eight samples were used for the transcriptome sequencing (Table 1). According to the differences in air temperature, the samples could be divided into five temperature groups: T2, T5, T10, T14 and T18 (Table 1).

The cDNA libraries were constructed from RNA samples for Illumina paired-end (PE) sequencing following the Illumina protocol. PE sequencing (2 × 100 bp) was carried out on the Illumina HiSeq 2000 platform (Illumina, San Diego CA, USA) at Novogene Bioinformatics Technology Co., Ltd (Beijing, China).

### Sequence assembly and Unigene annotation

Raw reads were processed to remove reads containing adaptors, with more than 10% ambiguous bases (N), or of low quality (more than 50% bases with small Q_phred_ ≤ 5). All the downstream analyses were based on the resulting clean reads. Clean reads were assembled using Trinity (version r2012-10-05) with min_kmer_cov = 2 [16]. The longest assembled transcript of a gene was taken as a unigene. All the assembled unigenes were used as reference sequences for the leaf transcriptome of wild oil-tea camellia.

Functional annotations of unigenes were based on the following databases: Nr (NCBI non-redundant protein sequences), Nt (NCBI non-redundant nucleotide sequences), Pfam (Protein family: http://pfam.xfam.org/) [17], KOG (euKaryotic Ortholog Groups)/COG (Clusters of Orthologous Groups of proteins) (http://www.ncbi.nlm.nih.gov/COG/) [18], Swiss-Prot (a manually annotated and reviewed protein sequence database: http://www.ebi.ac.uk/uniprot) [19], KEGG (Kyoto Encyclopedia of Genes and Genomes: http://www.genome.jp/kegg/) [20], and GO (Gene Ontology: http://geneontology.org/). NCBI blast 2.2.28+ was used for the alignments of unigenes to Nr, Nt, Swiss-Prot, and KOG. The E-value threshold was set to 1E − 5 in the alignments to Nr, Nt, and Swiss-Prot. For the alignments to KOG, the E-value threshold was 1E − 3. The hmmscan in HMMER 3.0 was used to search Pfam [21]. The GO annotations were performed with Blast2GO v2.5 [22] based on the Nr and Pfam annotations. KAAS (KEGG Automatic Annotation Server: http://www.genome.jp/kegg/kaas/) was used for the KEGG annotations [23].

### Detection of SSRs, SNPs and InDels

MISA 1.0 was used to detect SSRs in unigenes. The minimum repeat number for unit size of mono-, di-, tri-, tetra-, penta-, and hexanucleotide was set to 10, 6, 5, 5, 5, and 5, respectively. Primer3 (2.3.5) was used to design primers around SSRs with default settings.

Clean reads of each sample were aligned to the reference sequences (unigenes) using bowtie 2 (mismatch 0) [24]. The alignments were processed with SAMtools [25] and Picard tools for sequence sorting and duplicate removing. SNP and InDel callings were then performed using GATK2 [26]. Those with QUAL < 30.0 and QD < 5.0 were removed.

### Genetic structure analysis

In order to illustrate the genetic structure of wild oil-tea camellia samples from different latitudes and elevations, phylogenetic analysis was carried out based on the SNP data. SNP positions were chosen with no more than 2 alleles and the number of reads per sample ≥ 6. Then, an R [27] script was written to genotype each sample at each SNP position using IUPAC (International Union of Pure and Applied Chemistry) nucleic acid codes. Using the SNP genotypes of different samples, the Bayesian estimation of phylogeny was performed in MrBayes 3.2.5 by sampling across the entire general time reversible (GTR) model space [28]. The resulted consensus tree was viewed and edited in FigTree v1.4.2.

### Gene expression analysis

RSEM [29] was used to calculate the read count of each unigene in a sample and transform it into FPKM (expected number of fragments per kilobase of transcript per million fragments mapped) [30]. In RSEM analysis, bowtie was used with mismatch 2. The resulting FPKM values were used to represent the gene expression levels of unigenes in different samples. To examine whether sequencing depth was sufficient for gene expression analysis, varied percentages of mapped reads were randomly taken from each sample and fraction of genes with an expression level within 10% of the final expression value (according to 100% mapped reads) was calculated. A curve illustrating the relationship between fraction of genes within 10% of the final value and percentage of mapped reads was made for each sample. If a curve became flat (saturation) with the increase in percentage of mapped reads, the sequencing depth should be sufficient for gene expression analysis.

To examine the effects of air temperature on gene expression patterns of leaves, density distributions of FPKM values were compared among different temperature groups. Differential gene expression analyses between different temperature groups were performed using DESeq [31]. The threshold adjusted *p*-value for significance was 0.05. Hierarchical clustering and Venn diagram were used to illustrate the differential gene expression patterns between different temperature groups. According to the functional annotations of unigenes, putative functions of the differentially expressed genes (DEGs) were inferred to discover candidate genes for cold acclimation.

A new method was developed to figure out the major functional groups of genes involving in cold acclimation by comparing the GO annotations of DEGs with the GO annotations of all expressed genes detected in our study. To account for the effects of random sampling, all expressed genes were randomly sampled for 10000 times with a size equaling to the number of DEGs. Then, the number of DEGs in a GO functional group was tested for whether it was significantly different from the number of genes in the same GO functional group resulted from the random sampling of all expressed genes. The significant level was adjusted using the Bonferroni correction (0.05 divided by number of tests). An R script was written and used for the random sampling and statistical analysis.

### Quantitative real-time PCR analysis

In order to validate the DEGs identified from transcriptome sequencing, quantitative real-time PCR (qRT-PCR) analysis was performed. Independent wild oil-tea camellia leaf samples at different air temperatures were used: JG05 at 17.8 °C and JG06 at 14.7 °C from Jinggang Mountain; LS05 at 11.0 °C and LS06 at 4 °C from Lu Mountain. Total RNAs were extracted as described before for the samples used in transcriptome sequencing. Using the PrimeScript™ RT reagent qPCR Kit with gDNA Eraser (Takara, Dalian, China), genomic DNA was removed from total RNAs (300 ng RNAs of each sample) and cDNA was synthesized. The PCR mixture contained 12.5 μL SYBR® *Premix Ex Taq*™ II (Tli RNaseH Plus) (Takara, Dalian, China), 9.5 μL ddH_2_O, 1 μL of each gene-specific primer (10 μM) and 1 μL cDNA template. The qRT-PCR assays were performed in a CFX96 Touch™ RT-PCR Detection System (Bio-RAD, USA) with the following program: 94 °C for 2 min; 40 cycles of 94 °C for 20 s, 57 °C for 20 s and 72 °C for 30 s. A commonly used reference gene, glyceraldehyde-3-phosphate dehydrogenase (GAPDH) gene, was used to normalize the expression levels of target genes [32]. The relative expression levels of target genes were calculated with the 2^−∆∆Cq^ method [32].

## Results

### Summary of sequences and assembly

In total, 57.3 Gb high quality sequences were obtained from the transcriptome sequencing of wild oil-tea camellia leaves, ranging from 6.08 to 8.85 Gb per sample (Table 2). The average error rates of the sequences were 0.03–0.04% and more than 91% of the bases with error rates < 0.1% (Table 2). The sequencing data were assembled into 286121 transcripts with length ranging from 201 to 20507 bases (mean length = 708 bases and median length = 387 bases). As a result, 177258 unigenes were obtained (mean length = 517 bases and median length = 310 bases). The total length of the unigenes was 91.6 Mb (91556821 bases).

**Table 2:** Summary of the sequencing data from different wild oil-tea camellia samples

Sample	No. of clean reads	Clean bases (Gb)	Error rate (%)^a^	Q20 (%)^b^	Q30 (%)^c^	GC content (%)
LS01	69108798	6.91	0.04	96.02	91.08	45.68
LS02	77475252	7.75	0.04	96.70	91.92	46.18
LS03	60820248	6.08	0.04	96.12	91.17	45.85
LS04	63491250	6.35	0.03	97.74	92.96	48.14
JG01	88495706	8.85	0.04	96.26	91.30	45.92
JG02	70196006	7.02	0.03	97.99	93.24	47.72
JG03	70856840	7.08	0.04	96.23	91.04	46.06
JG04	72575848	7.26	0.04	96.42	91.46	46.52

^a^Percentage of the error bases

^b^Percentage of the bases with Q_phred_ > 20 (error rate < 1%)

^c^Percentage of the bases with Q_phred_ > 30 (error rate < 0.1%)

### Functional annotation of unigenes

**Fig. 1:**
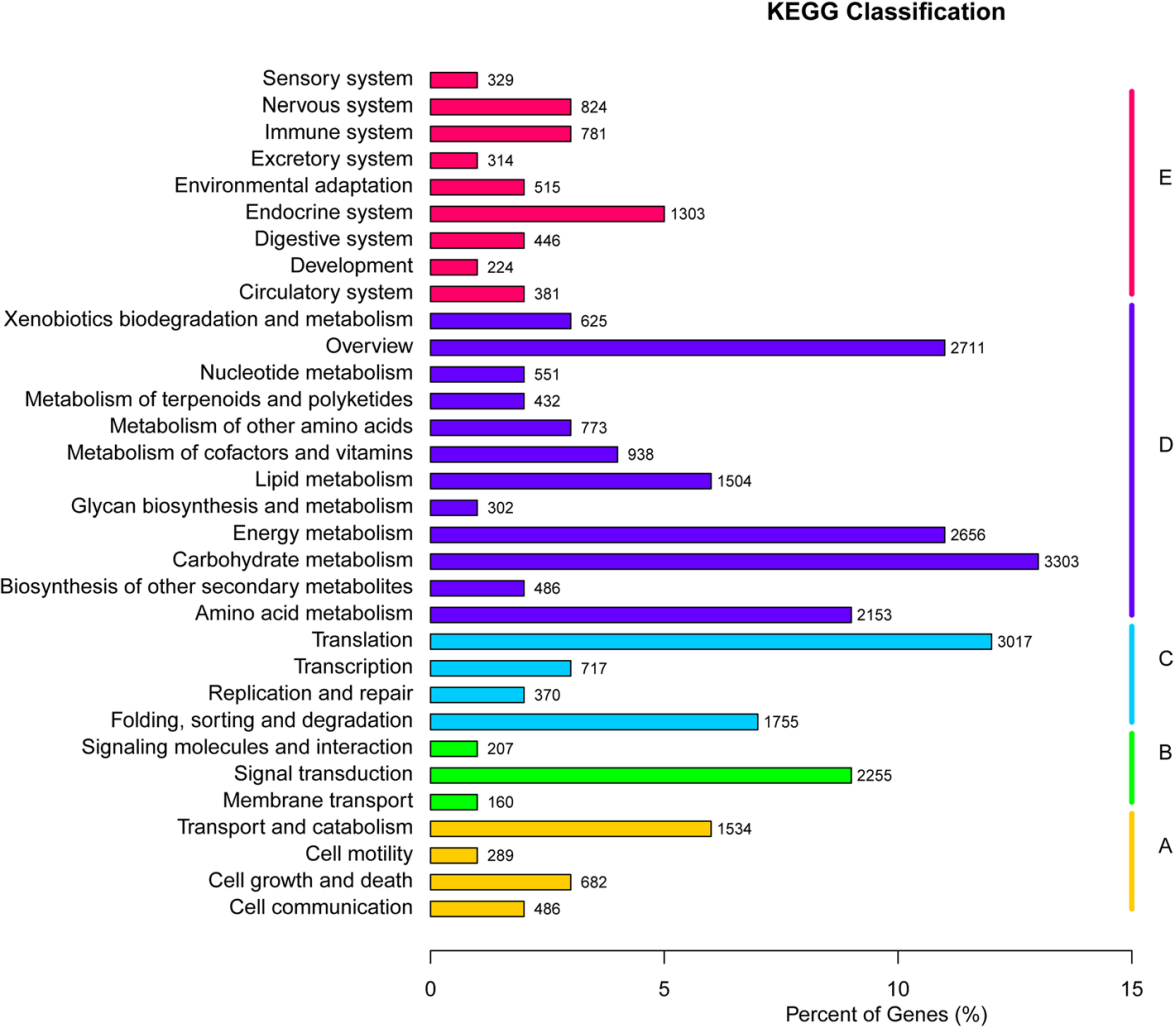
KEGG pathway classification of unigenes. *A* Cellular Processes, *B* Environmental Information Processing, *C* Genetic Information Processing, *D* Metabolism, and *E* Organismal Systems

In sum, 83352 unigenes (47.0% of the total unigenes) were annotated in at least one of the databases used in our study (Table 3). Those unigenes were mostly annotated in Nr database with good matches (best hits: median E-value = 3.6E − 36 and median Similarity = 0.82). According to the GO classification, the largest number of annotations was in Biological Process (BP), where the top 3 GO terms were cellular process, metabolic process and single-organism process; the second was Cellular Component (CC), where the top 3 were cell, cell part and organelle; the third was Molecular Function (MF), where the top 3 were binding, catalytic activity and transporter activity. For KOG classification, the top 10 classes were: (R) General function prediction only, (O) Posttranslational modification, protein turnover, chaperones, (J) Translation, ribosomal structure and biogenesis, (C) Energy production and conversion, (T) Signal transduction mechanisms, (G) Carbohydrate transport and metabolism, (U) Intracellular trafficking, secretion, and vesicular transport, (E) Amino acid transport and metabolism, (Q) Secondary metabolites biosynthesis, transport and catabolism, and (I) Lipid transport and metabolism. The results of KEGG pathway classification were shown in Fig. 1. The largest amount of the total annotations were involved in different metabolism pathways, among which carbohydrate metabolism was the most abundant following by the “overview” group, energy metabolism, amino acid metabolism and lipid metabolism etc.

**Table 3:** Annotation of unigenes in different databases

Database	No. of annotated unigenes	Percentage of annotated unigenes (%)
Nr	73520	41.5
GO	58275	32.9
Swiss-Prot	49605	28.0
Pfam	48178	27.2
Nt	28226	15.9
KOG	26601	15.0
KEGG	24659	13.9
At least one^a^	83352	47.0
All^b^	7394	4.2

^a^Annotated in at least one of the above databases

^b^Annotated in all of the above databases

### Detection of SSRs, SNPs and InDels

**Fig. 2:**
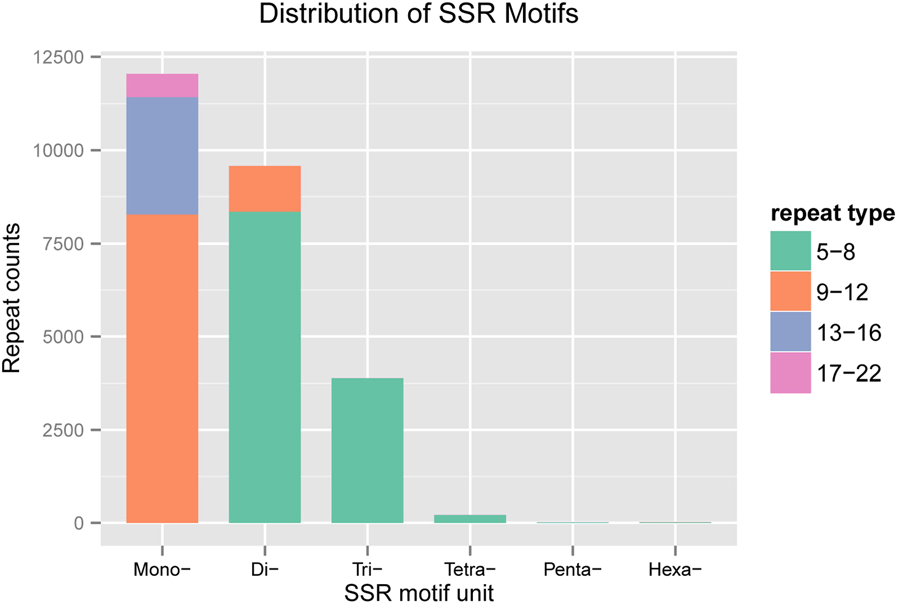
Distribution of SSR motifs. The x-axis indicates number of bases in SSR motif unit. The different color bars represent different repeat types (repeat number ranges of SSR motif unit)

We detected 25751 SSRs. The distribution of SSR motifs was shown in Fig. 2. About 46.8% of the SSRs were mononucleotide repeats, mainly of (A/T)_n_. The No. 2 SSRs were dinucleotide repeats (37.2%) and (AG/GA/CT/TC)_n_ was the most abundant dinucleotide repeats. With the increase in SSR motif unit size, the SSR abundance decreased dramatically (Fig. 2). Primers were successfully designed for 13962 SSRs. For the purposes of molecular marker development, complex SSRs [e.g. (TA)_6_(TAC)_6_, (CT)_8_tatct(TC)_6_] and those with a motif unit size less than two nucleotides were removed. As a result, 7005 SSR primers were obtained (Additional file 1: Table S1).

We discovered 661280 SNPs. About 54.3% of the SNPs were non-coding SNPs. For the coding SNPs, the ratio of non-synonymous to synonymous SNPs was 0.604, indicating most SNPs were synonymous. There were 103442 SNP positions with 2 alleles and number of reads ≥ 6 per sample (Additional file 2: Table S2). Genes containing the SNPs and ratio of non-synonymous to synonymous SNPs in each gene were summarized in Additional file 3: Table S3. Such data can help to develop SNP markers for oil-tea camellia.

We discovered 47056 InDels. For the purposes of molecular marker development, a gene containing only one InDel with two alleles was chosen and there were 6534 such InDels in total (Additional file 4: Table S4).

### Genetic structure

**Fig. 3:**
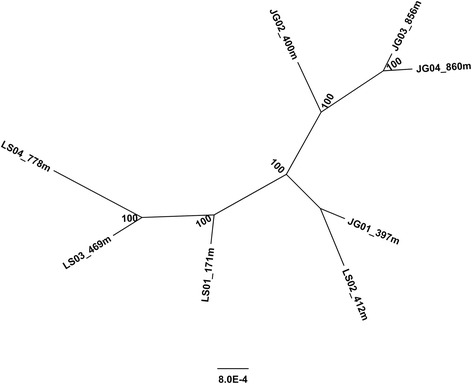
Phylogenetic tree of wild oil-tea camellia from different elevations in Lu and Jinggang Mountains. Tip labels indicate sample names and elevations. Those begin with “LS” are from Lu Mountain and “JG” from Jinggang Mountain. Node numbers indicate posterior probabilities (%)

We randomly chose 90000 SNP positions from the 103442 SNP positions (Additional file 2: Table S2) for the phylogenetic analysis. The phylogenetic tree constructed was illustrated in Fig. 3. Except for JG01 and LS02, wild oil-tea camellia samples from Lu and Jinggang Mountains were separated in the tree. Samples from higher elevations were genetically more differentiated between the two mountains.

### Differential gene expression

According to the relationships between fraction of genes within 10% of the final expression value (based on 100% mapped reads) and percentage of mapped reads, the curves became saturation when FPKM >3 for all samples (Additional file 5: Figure S1). Such results indicated that the sequencing depth was sufficient for gene expression analysis.

**Fig. 4:**
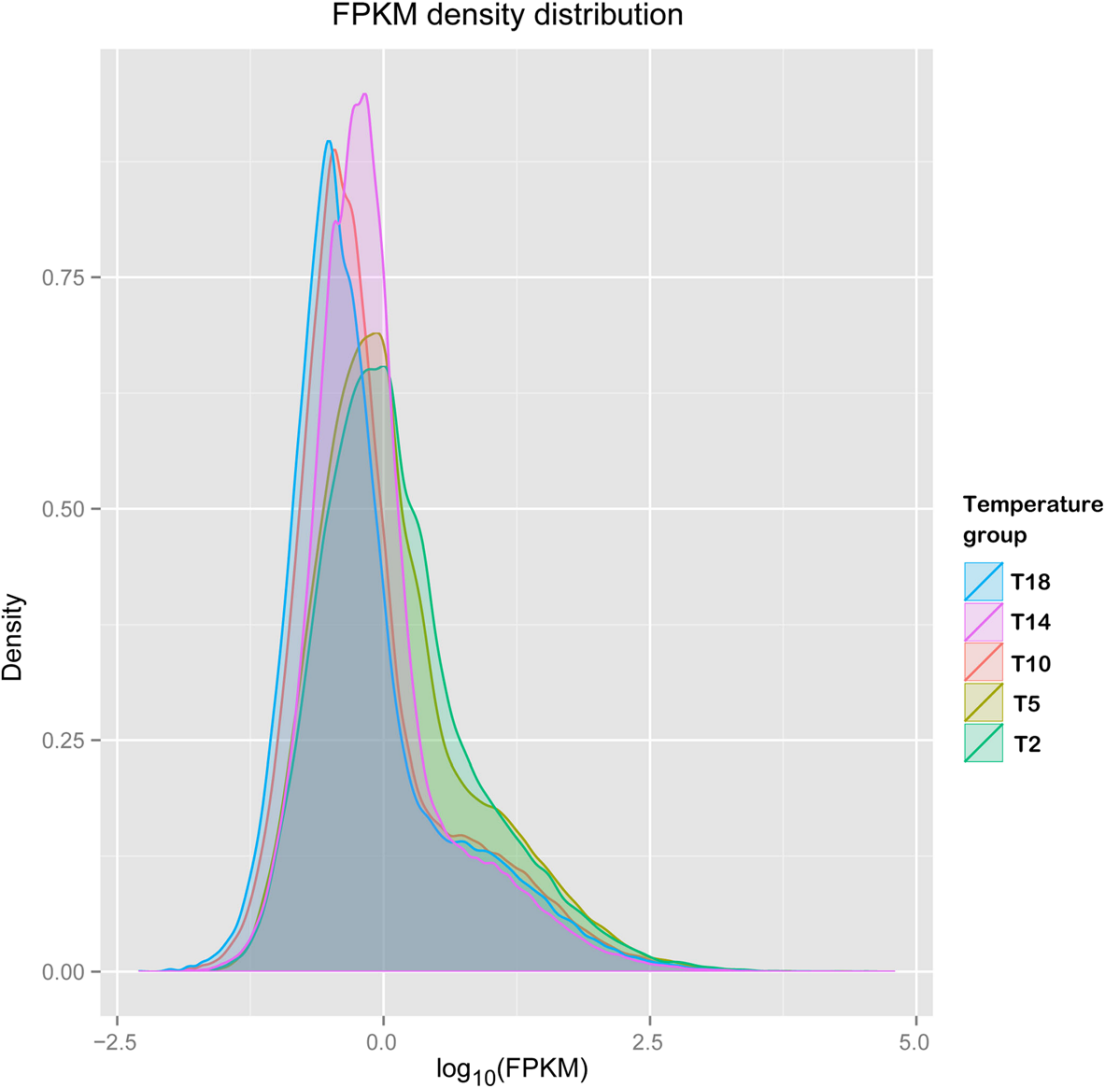
Density distribution of gene expression in different temperature groups. Gene expression levels are represented as log_10_(FPKM). See Table 1 for details of temperature groups

**Fig. 5:**
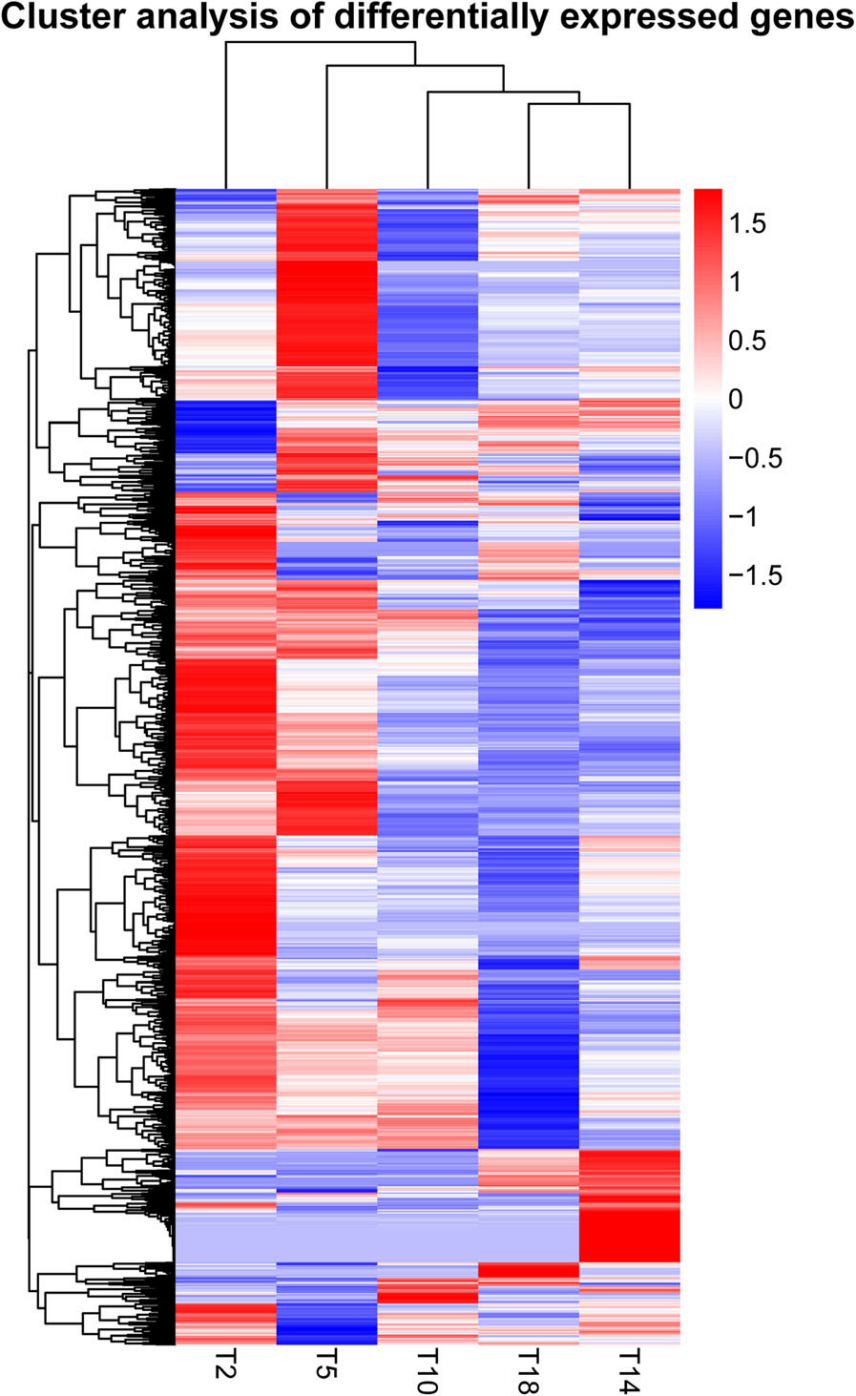
Hierarchical clustering heat map of differentially expressed genes. T2, T5, T10, T14 and T18 represent different temperature groups (different columns). A horizontal line shows the expression of a gene in different temperature groups. The expression of such a gene is significantly different in at least one of the pairwise comparisons between different temperature groups. Different colors indicate different levels of gene expression: from red to blue, the log_10_(FPKM + 1) value ranges from large to small

Density distributions of gene expression in different temperature groups were shown in Fig. 4. The gene expression patterns could be divided into two classes according to similarity: 1) T18, T14 and T10, representing relatively high air temperatures around 10–18 °C; and 2) T5 and T2, representing relatively low air temperatures around 2–5 °C. In general, when air temperature decreased to 2–5 °C, many genes had increased expression levels. Hierarchical clustering heat map of DEGs was illustrated in Fig. 5. In sum, T18 and T14 were clustered together indicating relatively high similarity in patterns of gene expression. Again, gene expression pattern altered with the decrease in air temperature. In particular, a considerable amount of genes were up-regulated at T5 and T2 showing similar gene expression patterns as indicated in Fig. 4. Moreover, the gene expression patterns were not exactly the same between T5 and T2 (Fig. 5).

**Fig. 6:**
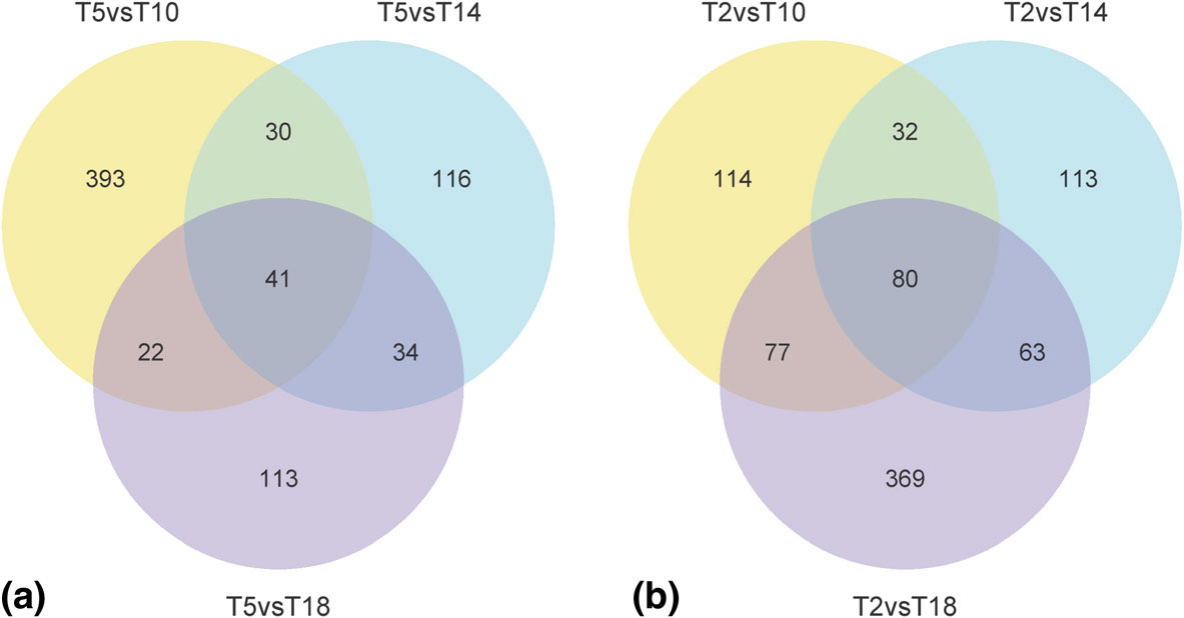
Venn diagrams of differentially expressed genes. **a** Number of differentially expressed genes in pairwise comparisons of gene expression between T5 and T10/T14/T18. **b** Number of differentially expressed genes in pairwise comparisons of gene expression between T2 and T10/T14/T18. Numbers in the overlapping regions refer to those genes differentially expressed in more than one pairwise comparison

Venn diagrams were used to summarize the number of DEGs between low and high air temperature groups (Fig. 6). Many genes were differentially expressed in only one or two of the pairwise comparisons. To identify candidate genes for cold acclimation with a low false-positive rate, close attention was paid to those genes showing consistent expression patterns at the low temperatures. 41 genes were differentially expressed in all the pairwise comparisons between T5 and T10/T14/T18 (Fig. 6a), where 40 genes were significantly up-regulated at T5 and only one gene (ID: comp196576_c0) was significantly down-regulated at T5 (Additional file 6: Table S5). 80 genes were differentially expressed in all the pairwise comparisons between T2 and T10/T14/T18 (Fig. 6b), and they were all significantly up-regulated at T2 (Additional file 7: Table S6). Among the 80 genes, 60 were differentially expressed between T2 and T5 (all significantly up-regulated at T2; Additional file 7: Table S6). Compared to T10/T14/T18, only 5 genes (ID: comp208485_c0, comp210221_c1, comp214280_c0, comp218213_c0 and comp220377_c0) were significantly up-regulated at both T5 and T2, where 2 genes were differentially expressed between T2 and T5 (comp210221_c1 and comp218213_c0). Such results indicated that the responses of gene expressions at T2 were different from those at T5 suggesting an increase in low temperature stress at T2. SNPs were found in 5 DEGs at T5 (Additional file 8: Table S7). Ratio of non-synonymous to synonymous SNPs was no more than 1 in these DEGs (0 in 2 DEGs and 0.833–1 in 3 DEGs). SNPs were found in 25 DEGs at T2 (Additional file 8: Table S7). Ratio of non-synonymous to synonymous SNPs was 0 in 13 DEGs, 0.333–0.5 in 3 DEGs, 0.875–1 in 3 DEGs and ≥2 in 6 DEGs. Such results implied that most of the DEGs might be under purifying selection. A few DEGs at T2 with the ratio of non-synonymous to synonymous SNPs ≥2 might be under positive selection.

**Fig. 7:**
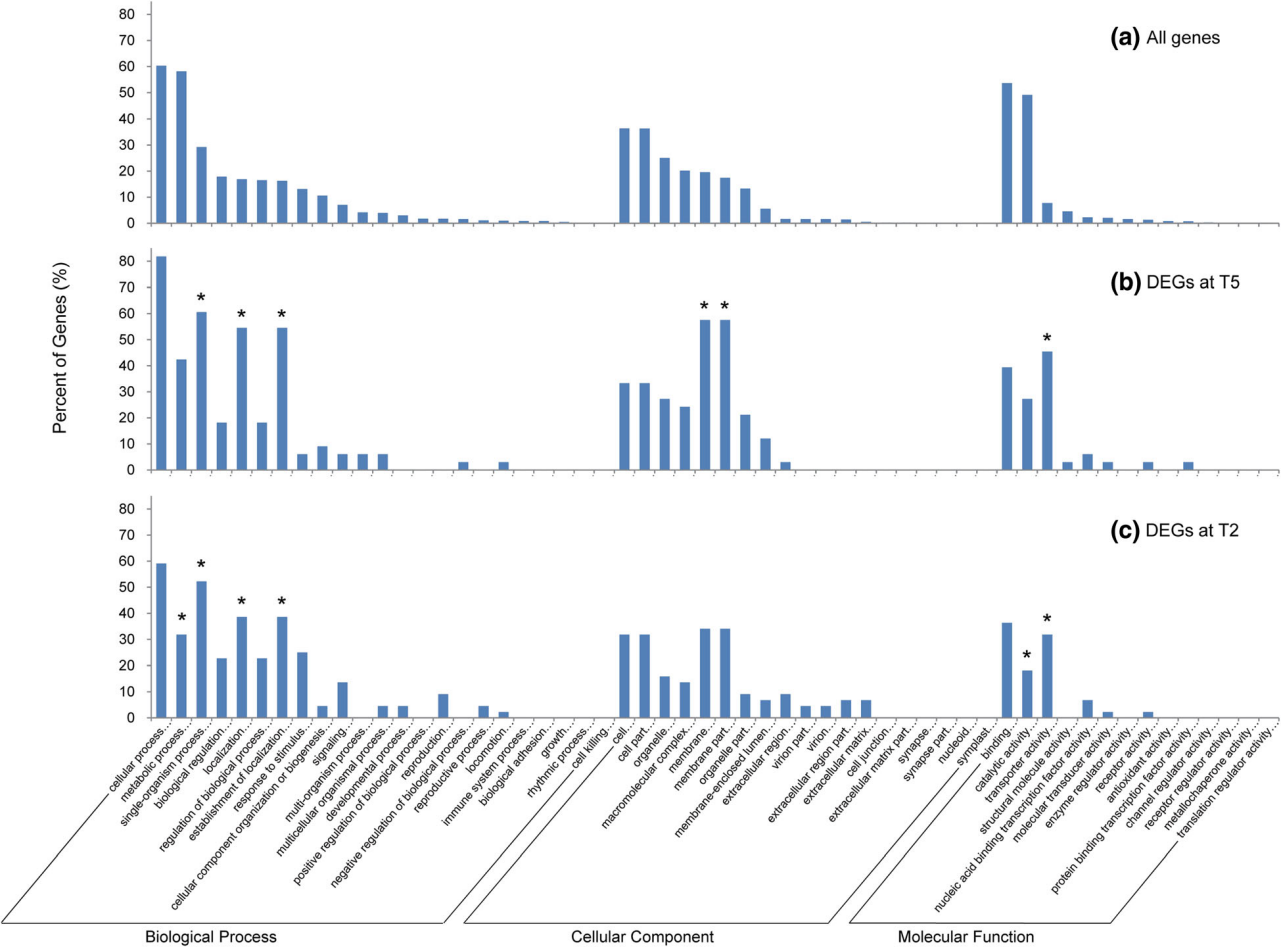
GO classification of genes. **a** GO classification of all expressed genes detected in our study including all temperature groups (T2, T5, T10, T14 and T18). **b** GO classification of differentially expressed genes (DEGs) at T5 versus T10/T14/T18. **c** GO classification of DEGs at T2 versus T10/T14/T18. Stars above bars indicate the amounts of differentially expressed genes are significantly higher or lower than the amounts of genes in random samples from the GO classification of all genes

For the DEGs at T5, 33 were annotated in the GO database. The percent of genes in different functional groups were shown in Fig. 7. Compared to the GO annotation of all genes, significantly higher amounts of the DEGs were annotated in the biological processes of single-organism process, localization and establishment of localization, the cellular components of membrane and membrane part, and the molecular function of transporter activity (Fig. 7). Collectively, these corresponded to the products of 15 genes, which were integral components of membranes and had transmembrane transporter activities (Table 4). All the genes were significantly up-regulated at T5. Among these gene products, 11 belonged to sugar transporters (Table 4). For the DEGs at T2, 44 were annotated in the GO database. The distribution of genes in different functional groups was quite similar to that at T5 (Fig. 7). Compared to the GO annotation of all genes, significantly higher amounts of the DEGs were annotated in the biological processes of single-organism process, localization and establishment of localization, and the molecular function of transporter activity (Fig. 7). These corresponded to the products of 14 genes, which were integral components of membranes and had transmembrane transporter activities (Table 4). All the genes were significantly up-regulated at T2. 8 were sugar transporter genes and 3 of the genes also significantly up-regulated at T5 (Table 4). Significantly lower amounts of DEGs at T2 were annotated in the biological process of metabolic process and the molecular function of catalytic activity (Fig. 7). A putative C-repeat binding factor (CBF) gene (ID: comp188417_c0) was significantly up-regulated at T2, which may be related to the biological process of cold acclimation (Additional file 7: Table S6).

**Table 4:** Significant functional groups of differentially expressed genes (DEGs) at T5/T2 versus T10/T14/T18. All products of the DEGs are integral components of membranes with transmembrane transporter activities

Putative function	DEGs at T5^a^	DEGs at T2
Sugar transporter	comp200120_c0, comp203054_c0, comp206804_c0, comp209071_c1, comp212144_c0, comp212939_c0, **comp214280_c0**, comp215715_c0, comp216420_c0, **comp218213_c0**, **comp220377_c0**	comp183939_c0, comp207638_c0, comp209330_c0, comp214001_c0, **comp214280_c0**, comp215310_c0, **comp218213_c0**, **comp220377_c0**
Amino acid transporter	comp211818_c0	comp208433_c0
Myo-inositol transporter	comp193115_c1, comp199501_c0, comp217974_c0	−
Sodium/calcium exchanger	−	comp167153_c0
Small conductance calcium-activated potassium channel	−	comp216277_c0
Dicarboxylic acid transmembrane transporter	−	comp185710_c0
ATPase coupled transporter	−	comp213346_c0
ATP synthase coupled hydrogen ion transporter	−	comp203688_c0

^a^Gene IDs in bold indicate DEGs at both T5 and T2

### Quantitative real-time PCR analysis

**Fig. 8:**
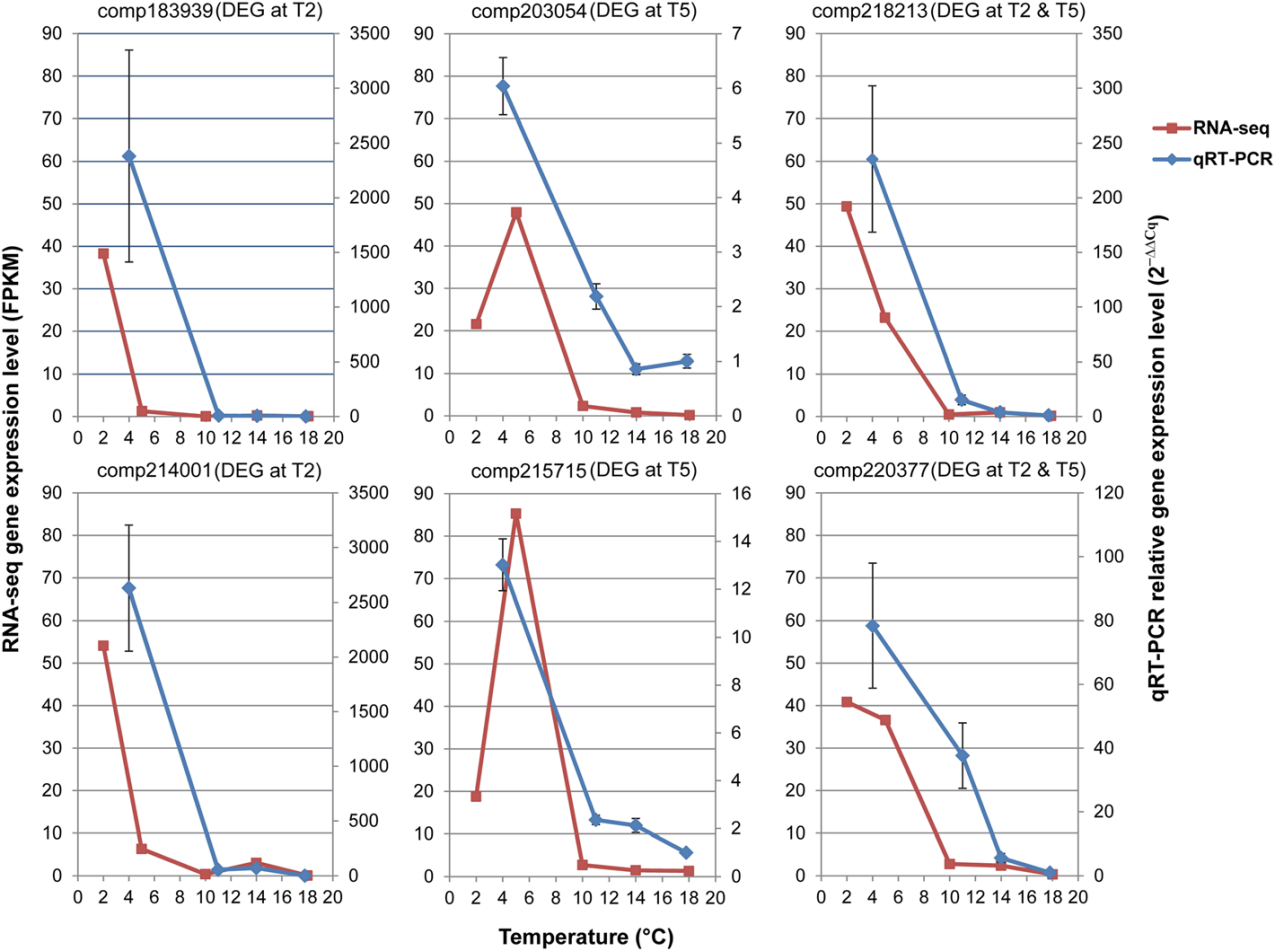
Gene expression levels of differentially expressed genes (DEGs) through RNA-seq and qRT-PCR analyses. All the genes are sugar transporter genes

Sugar transporter genes differentially expressed at T5 or/and T2 in RNA-seq (Table 4) were chosen for qRT-PCR analysis. Among these genes, 7 genes were excluded for relatively low expression levels (FPKM < 18) at all temperatures. Among the other 9 genes, 3 genes had unspecific amplifications in PCRs. At last, 6 genes were used for qRT-PCR analysis (Additional file 9: Table S8). The qRT-PCR analysis showed that the relative expression levels of all 6 genes dramatically increased at 4 °C (Fig. 8). The expression patterns detected in qRT-PCR fit well with those in RNA-seq analysis (Fig. 8). Such results demonstrated that DEGs identified based on transcriptome sequencing were reliable.

## Discussion

### Leaf transcriptome of wild oil-tea camellia

Our study obtained a high-quality and large transcriptome dataset of wild oil-tea camellia, representing the leaf transcriptome variation from different latitudes and elevations. The amount of data obtained (57.3 Gb), the number (177258) and the total length (91.6 Mb) of unigenes assembled are all much larger than those reported in a previous study on the transcriptome of oil-tea camellia (170 Mb data and 104842 unigenes with a total length of 38.9 Mb) by Xia et al. (2014) [15]. Such differences may be mainly due to the fact that the Illumina Hiseq 2000 high-throughput sequencing platform used in our study can produce a much larger amount of sequencing data than the 454 GS-FLX sequencing platform applied in the previous study. In our study, 83352 unigenes were annotated (Table 3), and 1504 unigenes were found to be involved in the lipid metabolism pathways (Fig. 1), which can serve as a basis for understanding the processes of fatty-acid biosynthesis in this important oil crop. Moreover, our study used more diverse samples of wild oil-tea camellia from various latitudes and elevations, and therefore the SSRs, SNPs and InDels identified in our study may be more useful for developing molecular markers to detect the genetic structure of wild oil-tea camellia along latitude and elevation gradients.

The genus *Camellia* has about 120 species. Besides of oil-tea camellia, the genus *Camellia* has many other important economic species. Tea plant, *C. sinensis*, is one of the most important economic crops, generating the most popular non-alcoholic beverage in the world. Tea plant is also the best-studied species in the genus *Camellia* with the largest amount of genomic data available in public databases. A deep transcriptome sequencing of tea plant was published in 2011, where 2.32 Gb high-quality data were generated and assembled into 127094 unigenes with a total length of 45.1 Mb [33]. Wang *et al.* [34] reported the leaf transcriptomes of tea plant in response to cold acclimation. They obtained 4.96 Gb high-quality sequencing data and assembled 216831 non-redundant transcript sequences with a total length of 77.4 Mb [34]. After combining all available transcriptome data of the tea plant, they got 282395 non-redundant transcript sequences with a total length of 94.7 Mb. Therefore, the transcriptome sequencing data of oil-tea camellia obtained in our study also provide one of the largest transcriptome dataset in the genus *Camellia*. Such a large transcriptome dataset of oil-tea camellia can facilitate the functional genomic studies and the molecular breeding of many other economically important *Camellia* species, especially for those more closely related to oil-tea camellia (subgenus Camellia) than tea plant (subgenus Thea), such as the famous ornamental plants *C. japonica* and *C. sasanqua*.

### Genetic structure of wild oil-tea camellia

Based on 90000 SNPs, a phylogenetic tree was constructed with the wild oil-tea camellia samples from different latitudes and elevations. The wild oil-tea camellia samples seem to be separated mainly by latitudes (between Lu and Jinggang Mountains) and then by elevations (within Lu or Jinggang Mountain) (Fig. 3). Previous studies indicated that air temperature was one of the major factors affecting the growth and development of oil-tea camellia, which may lead to genetic differentiation along air temperature gradients [6]. Because air temperature decreases 1 °C with an increase of about 167 m in elevation or about 145 km in latitude, gene flow may be more restricted between different latitudes than between different elevations along the same air temperature gradients [35]. Thus, genetic differentiation may become more distinct between different latitudes than between different elevations [35]. However, the genetic differentiation of wild oil-tea camellia between Lu and Jinggang Mountains is incomplete (Fig. 3). Wild oil-tea camellia sample LS02 from Lu Mountain is grouped together with JG01 from Jinggang Mountain. Such results suggest that wild oil-tea camellia from the two mountains may be isolated recently so that the effects of genetic drift and natural selection could not completely drive them apart to form two distinct branches. Although the genetic structure was constructed on a huge amount of SNPs, only eight wild oil-tea camellia samples from two mountains were used in our study. A large number of wild oil-tea camellia samples from various latitudes and elevations should be used to exactly identify the genetic structure in future studies.

### Differential gene expressions under cold acclimation

Tropical or subtropical plants may experience chilling stress when air temperatures fall below 10 °C [1]. A previous study also showed that tea plant (*C. sinensis*) underwent cold acclimation when air temperatures were below 10 °C [34]. Many genes were differentially expressed in tea plant leaves during the cold acclimation process, and the number of up-regulated genes was about twice the number of down-regulated genes [34]. In our study, the gene expression patterns in the leaves of wild oil-tea camellia obviously changed when air temperatures were below 10 °C (Fig. 4), suggesting that oil-tea camellia may also undergo cold acclimation when air temperature was below 10 °C. Again, almost all the DEGs at T2 and T5 were significantly up-regulated, showing that most of the genes were activated instead of being repressed during the cold acclimation process as also indicated in the above study of tea plant. In addition, more genes in oil-tea camellia leaves were differentially expressed at T2 than at T5, suggesting a stronger cold stress at T2. This may be due to the fact that wild oil-tea camellia underwent a longer period at lower air temperatures in the habitats of higher elevations.

Among the DEGs in cold acclimation, transmembrane transporter genes were found to be predominant in oil-tea camellia leaves. Many of the genes encode transmembrane sugar transporters (Table 4). Researches on tea plants, another *Camellia* species, also found that sugar transporter genes were differentially expressed in leaves during the cold acclimation process and may lead to sugar accumulation in cells [34, 36]. During the cold acclimation process, the soluble sugar content was found to be constantly elevated in leaves of tea plants [36]. In addition, amino acid transporters were found to be significantly up-regulated in oil-tea camellia leaves at both T2 and T5, which may lead to amino acid accumulation in cells (Table 4). The accumulation of soluble sugars and amino acids in cells is thought to be one of the most predominantly metabolic changes in many plant species during cold acclimation [4, 36–39]. The massive increase of such solutes may help to adjust osmotic pressure in cells to decrease freezing temperature of cell sap and protect membranes against freeze-induced damages [40]. Further metabolic studies are needed to find out whether or not soluble sugars and amino acids do increase in oil-tea camellia leaves during cold acclimation.

On the other hand, five transporter genes were found to be significantly up-regulated in oil-tea camellia leaves only at T2 (Table 4). Among the products of these genes, putative sodium/calcium exchanger and small conductance calcium-activated potassium channel may be related to the calcium signaling pathway. Calcium is an important second messenger in the low-temperature signal transduction pathway regulating the cold-acclimation response [4]. Most of the genes in the calcium signaling pathway were also found to be up-regulated in tea plants during cold acclimation [34]. In addition, putative sodium/calcium exchanger and dicarboxylic acid transmembrane transporter were found to increase in leaf tonoplasts of *Arabidopsis thaliana* upon cold acclimation [39]. Therefore, the transporter genes only up-regulated in oil-tea camellia leaves at T2 may also be owing to the effects of cold acclimation. Moreover, a putative C-repeat binding factor (CBF) gene was significantly up-regulated in oil-tea camellia leaves only at T2. The CBFs are the major transcriptome factors regulating the expression of a large number of genes in cold acclimation [41]. The CBF gene expression can be activated in about 15 min after transferring plants to low temperature (4 °C) [41]. The significant up-regulation of genes concerned with cold acclimation in oil-tea camellia leaves only at T2 suggests that the air temperature at T5 may not be sufficiently low to remarkably activate the expressions of those genes.

Differential gene expression can be due to differences in genotype, environmental condition and the interaction between them. According to the genetic structure of wild oil-tea camellia (Fig. 3), most of the genetic differentiation occurred between Lu and Jinggang Mountains. However, the gene expression patterns could not be well explained by such a genetic structure. Sharp changes were observed in gene expressions of wild oil-tea camellia samples within the Lu Mountain instead of between the two mountains, and the gene expression patterns of two samples (T10: LS01 and LS02) from the Lu Mountain was similar to those (T14 and T18) from the Jinggang Mountain (Figs. 4 and 5). Meanwhile, the gene expression patterns could be well explained by the differences in air temperature. In general, when air temperature decreased to 2–5 °C (T2 and T5) from around 10–18 °C (T10, T14 and T18), many genes had increased expression levels. To minimize the random effects on gene expression of environmental factors other than air temperature and the interactions with genotypes, we selected genes showing consistently differential expression patterns between low (T2 or T5) and high (T10, T14 or T18) temperatures. The distributions of these DEGs in different functional groups are quite similar between T2 and T5 (Fig. 7), demonstrating that the differential gene expression patterns are not due to random effects. The predominantly functional groups of the DEGs were determined, and most of the genes were also reported to be differentially expressed during cold acclimation in previous studies. The qRT-PCR analysis using independent wild oil-tea camellia leaf samples at different air temperatures showed that the relative expression levels of the selected sugar transporter genes dramatically increased at 4 °C (Fig. 8). Such results demonstrated that the DEGs identified were reliable. Thus, we can conclude that the differential gene expressions in wild oil-tea camellia leaves observed in our study may be mainly due to the differences in air temperature. The DEGs identified may represent the major candidate genes concerned with the cold acclimation process in oil-tea camellia.

## Conclusions

The leaf transcriptomes of wild oil-tea camellia obtained in our study provide one of the largest transcriptome dataset in the genus *Camellia*. Such a dataset can facilitate the functional genomic studies and the molecular breeding of many economically important *Camellia* species. Large amounts of SSRs, SNPs and InDels were identified. The phylogenetic analysis based on SNPs showed genetic differentiation between latitudes. Such a result suggests that the sequence variations identified can be used to develop molecular markers for analyzing genetic differentiations along latitude and elevation gradients in wild oil-tea camellia. Wild oil-tea camellia may undergo cold acclimation when air temperatures are below 10 °C. During cold acclimation, many genes may be up-regulated and the number of DEGs increases with the decrease in air temperature. We provide a new method for identifying significant functional groups of DEGs. Based on the new method, our study clearly showed that candidate genes for cold acclimation may be predominantly involved in transmembrane transporter activities. Our study can serve as a basis for studying molecular mechanisms of cold tolerance in evergreen broadleaf woody plants.

## Additional files

Additional file 1: Table S1. Primers designed for detecting simple sequence repeats (SSRs) in genes of *Camellia oleifera*. (XLSX 3329 kb)
Additional file 2: Table S2. Single nucleotide polymorphism (SNP) positions in genes of *Camellia oleifera*. Genotypes of samples from Jinggang (JG01-04) and Lu (LS01-04) mountains are shown. (XLSX 8324 kb)
Additional file 3: Table S3. Genes containing single nucleotide polymorphisms (SNPs). Number of non-synonymous SNPs (N), synonymous SNPs (S) and ratio between them (N/S) are shown. (XLSX 8950 kb)
Additional file 4: Table S4. Genes containing insertion/deletions (InDels) in *Camellia oleifera*. (XLSX 3536 kb)
Additional file 5: Figure S1. Relationships between fraction of genes within 10% of the final expression value (according to 100% mapped reads) and percentage of mapped reads. (DOC 230 kb)
Additional file 6: Table S5. Differentially expressed genes at T5 versus T10/T14/T18, showing gene sequences, gene expression levels in different temperature groups and details of functional annotations. (XLSX 46 kb)
Additional file 7: Table S6. Differentially expressed genes at T2 versus T10/T14/T18, showing gene sequences, gene expression levels in different temperature groups and details of functional annotations. (XLSX 64 kb)
Additional file 8: Table S7. Number of SNPs, number of non-synonymous SNPs (N), number of synonymous SNPs (S) and N/S in differentially expressed genes (DEGs) at T5 and T2. (DOC 47 kb)
Additional file 9: Table S8. Genes and primers used for qRT-PCR analysis. (DOC 33 kb)
