# Supplementary material for: Leaf transcriptome analysis of a subtropical evergreen broadleaf plant, wild oil-tea camellia (Camellia oleifera), revealing candidate genes for cold acclimation
Source: BMC Genomics. 2017 Feb 28;18:211. doi: 10.1186/s12864-017-3570-4 (PMC5329932; doi:10.1186/s12864-017-3570-4)
Supplement: Additional file 5: Figure S1. — Relationships between fraction of genes within 10% of the final expression value (according to 100% mapped reads) and percentage of mapped reads. (DOC 230 kb) [file 12864_2017_3570_MOESM5_ESM.doc]

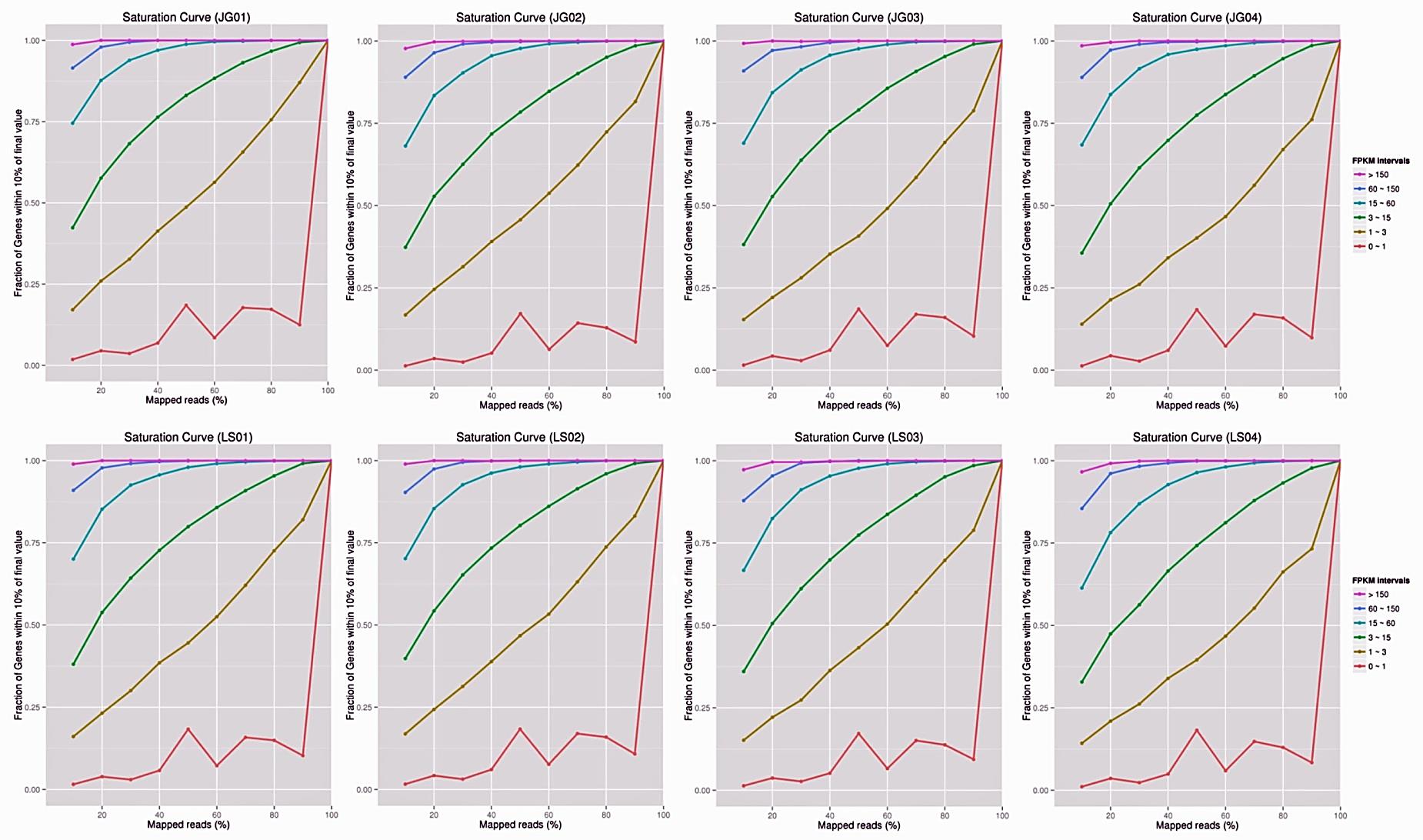


**Fig. S1** Relationships between fraction of genes within 10% of the final expression value (according to 100% mapped reads) and percentage of mapped reads. JG01-04 indicate samples from Jinggang Mountain and LS01-04 represent samples from Lu Mountain. Curves with different colours represent different FPKM intervals.
