# Supplementary material for: Leaf transcriptome analysis of a subtropical evergreen broadleaf plant, wild oil-tea camellia (Camellia oleifera), revealing candidate genes for cold acclimation
Source: BMC Genomics. 2017 Feb 28;18:211. doi: 10.1186/s12864-017-3570-4 (PMC5329932; doi:10.1186/s12864-017-3570-4)
Supplement: Additional file 8: Table S7. — Number of SNPs, number of non-synonymous SNPs (N), number of synonymous SNPs (S) and N/S in differentially expressed genes (DEGs) at T5 and T2. (DOC 47 kb) [file 12864_2017_3570_MOESM8_ESM.doc]

**Table S7** Number of SNPs, number of non-synonymous SNPs (N), number of synonymous SNPs (S) and N/S in differentially expressed genes (DEGs) at T5 and T2. Genes with N/S > 1 are indicated in bold.

| Gene ID | Number of SNPs | N | S | N/S |
| --- | --- | --- | --- | --- |
| *DEGs at T5* |  |  |  |  |
| comp179202_c0 | 2 | 0 | 2 | 0 |
| comp201314_c0 | 4 | 2 | 2 | 1 |
| comp202187_c0 | 11 | 5 | 6 | 0.833 |
| comp210221_c1 | 15 | 7 | 8 | 0.875 |
| comp217139_c0 | 2 | 0 | 2 | 0 |
| *DEGs at T2* |  |  |  |  |
| comp128704_c0 | 2 | 0 | 2 | 0 |
| **comp185710_c0** | **4** | **3** | **1** | **3** |
| comp187389_c0 | 1 | 0 | 1 | 0 |
| comp192253_c0 | 6 | 0 | 6 | 0 |
| **comp194867_c0** | **4** | **3** | **1** | **3** |
| **comp196533_c0** | **6** | **4** | **2** | **2** |
| comp199080_c1 | 1 | 0 | 1 | 0 |
| comp199532_c0 | 4 | 0 | 4 | 0 |
| comp200536_c0 | 4 | 1 | 3 | 0.333 |
| comp200771_c0 | 3 | 1 | 2 | 0.5 |
| comp201126_c0 | 3 | 0 | 3 | 0 |
| comp201496_c0 | 3 | 0 | 3 | 0 |
| comp205356_c0 | 6 | 0 | 6 | 0 |
| comp207017_c0 | 2 | 0 | 2 | 0 |
| comp208850_c0 | 4 | 2 | 2 | 1 |
| comp209420_c0 | 9 | 0 | 9 | 0 |
| comp210221_c1 | 15 | 7 | 8 | 0.875 |
| **comp210485_c0** | **8** | **7** | **1** | **7** |
| comp212053_c2 | 14 | 4 | 10 | 0.4 |
| comp212682_c0 | 10 | 0 | 10 | 0 |
| comp213346_c0 | 6 | 3 | 3 | 1 |
| comp216277_c0 | 3 | 0 | 3 | 0 |
| **comp216763_c1** | **2** | **2** | **0** | **Inf** |
| comp217071_c0 | 2 | 0 | 2 | 0 |
| **comp74978_c0** | **2** | **2** | **0** | **Inf** |
