# Supplementary material for: Leaf transcriptome analysis of a subtropical evergreen broadleaf plant, wild oil-tea camellia (Camellia oleifera), revealing candidate genes for cold acclimation
Source: BMC Genomics. 2017 Feb 28;18:211. doi: 10.1186/s12864-017-3570-4 (PMC5329932; doi:10.1186/s12864-017-3570-4)
Supplement: Additional file 9: Table S8. — Genes and primers used for qRT-PCR analysis. (DOC 33 kb) [file 12864_2017_3570_MOESM9_ESM.doc]

**Table S8** Genes and primers used for qRT-PCR analysis.

| **Gene ID** | **Forward (F) and Rewards (R) Primers** | **PCR Products Size（bp）** |
| --- | --- | --- |
| **Sugar transporter gene** | | |
| comp218213_c0 | F: 5’-CATCTCCTTCGGTGGTGTCCTG-3’  R: 5’-GGCGGACAGGATAGACACGATC-3’ | 169 |
| comp220377_c0 | F: 5’-CAGACCTTGACCACCACCTACG-3’  R: 5’-TTAAGACACCAGCGGCGATCAA-3’ | 118 |
| comp183939_c0 | F: 5’-TGACCGAGTACCTGAAGACCCA-3’  R: 5’-CTAGCACTTCCCCGAACACCTC-3’ | 121 |
| comp214001_c0 | F: 5’-TATGCCTCAGAGATTTGCCCCG-3’  R: 5’-TAAGAAGACCTCGCAGCACACC-3’ | 112 |
| comp203054_c0 | F: 5’-AGACTTTGACCACCACCTACGC-3’  R: 5’-TTCAGAACACCAGCGGCGATTA-3’ | 118 |
| comp215715_c0 | F: 5’-ACACCAGCTACCACATTGCCAT-3’  R: 5’-AATGATGGTTCCACGTCGCTCA-3’ | 155 |
| **Reference gene** | | |
| GAPDH | F: 5’-TTGGCATCGTTGAGGGTCT-3’  R: 5’-CAGTGGGAACACGGAAAGC-3’ | 206 |
